# p53 Upregulation Is a Frequent Response to Deficiency of Cell-Essential Genes

**DOI:** 10.1371/journal.pone.0015938

**Published:** 2010-12-31

**Authors:** Nadia Danilova, Asako Kumagai, Jenny Lin

**Affiliations:** Department of Molecular, Cell and Developmental Biology, University of California Los Angeles, Los Angeles, California, United States of America; Roswell Park Cancer Institute, United States of America

## Abstract

**Background:**

The role of p53 in the prevention of development of embryos damaged by genotoxic factors is well recognized. However, whether p53 plays an analogous role in preventing birth defects from genetic mutations remains an unanswered question. Genetic screens for mutations affecting development show that only a fraction of developmentally lethal mutations leads to specific phenotypes while the majority results in similar recurrent phenotypes characterized by neuronal apoptosis and developmental delay. Mutations in cell-essential genes typically fall into this group. The observation that mutations in diverse housekeeping genes lead to a similar phenotype suggests a common mechanism underlying this phenotype. For some mutants, p53 inhibition was shown to attenuate the phenotype.

**Methodology/Principal Findings:**

To find out how common p53 involvement is in this phenotype, we analyzed zebrafish mutants from various categories of cell essential genes. Several thousand zebrafish mutants have been identified; many of them are kept at stock centers and available for the research community. We selected mutants for genes functioning in DNA replication, transcription, telomere maintenance, ribosome biogenesis, splicing, chaperoning, endocytosis, and cellular transport. We found that mutants have similar phenotypes including neural apoptosis, failure to develop structures originated from the neural crest cells, and hematopoietic defects. All mutants share p53 upregulation and similar changes in several p53-dependent and independent molecular pathways.

**Conclusion/Significance:**

Our results suggest that mutations in housekeeping genes often canalize on the p53-mediated phenotype. p53 prevents the development of embryos with defects in such genes. p53-mediated changes in gene expression may also contribute to many human congenital malformations.

## Introduction

Some time ago, Hall and Lane suggested that the ability of p53 to prevent teratogenesis was the primary driving force behind the evolution of this protein mainly known as a tumor suppressor [1]. The “guardian of the babies” idea was inspired by several reports indicating that p53 decreases the level of congenital abnormalities in experimental animals exposed to drugs or radiation [2], [3]. For example, X-ray irradiation of pregnant wild type mice leads to a 20% incidence of anomalies and a 60% incidence of death in embryos, while in *p53*−/− mice the incidence of anomalies increases to 70% and the frequency of death decreases to 7% [3]. Thus, activation of p53 in response to genotoxic stress promotes the death of damaged embryos, and in this way, decreases the incidence of congenital defects among the survivors.

In humans, about half of all pregnancies end in spontaneous abortions early in development, while in mice the incidence of sporadic death is 20–25% [4]. Embryonic death can be caused by both environmental and genetic factors. The results of mutagenesis screens suggest that disruption of 1,400 to 2,400 genes could lead to early embryonic death in zebrafish [5]. These development-essential genes tend to be evolutionarily conserved.

Of the mutations affecting zebrafish development, only about one-third leads to relatively specific developmental defects while the majority lead to more general and recurring phenotypes manifested by increased neuronal necrosis/apoptosis and developmental delay. For this reason, of the more than 6,600 mutations identified in mutagenesis screens in 1996, only 1740 were maintained while others were discarded as “non-specific” [6], [7]. In the screen performed at the Hopkins' lab, all mutants with visible embryonic phenotypes were maintained regardless of specificity [5]. Therefore, these mutants represent a valuable recourse to study the mechanisms underlying the non-specific phenotype.

From mutants in the above study about half had non-specific phenotype with neuronal apoptosis. In most cases, this phenotype was associated with a mutation in a housekeeping gene. Next, we analyzed the status of the p53 network in randomly selected mutants from various categories of housekeeping genes and found that they all had p53 upregulation. p53 upregulation was especially pronounced in neural cells and p53 inhibition reduced neuronal apoptosis. The mutants with upregulated p53 network had similar changes in several p53-dependent and independent molecular pathways. Therefore, p53-mediated changes may underlie the common phenotype of mutants for cell-essential genes.

p53 may provide a checkpoint for development by integrating genetic and environmental information. It prevents the development of defective embryos beyond the early stages, thereby preserving genomes at the population level.

## Results

### Mutants for cell-essential genes have similar phenotypes

Among 315 mutants identified in the Hopkins' screen (see supplemental table 3 in [5]), 157 have a similar phenotypic signature, characterized by various degrees of brain necrosis at day one, and at later stages, by a smaller head and eyes and developmental delay. Analysis of genes affected in these mutants showed that they encode proteins involved in ribosome biogenesis (31%), chromatin maintenance, DNA replication and repair (17%), transcription (9%), mRNA processing (14%), protein folding and degradation, and other basic cellular functions. The observation that mutations in diverse housekeeping genes lead to a similar phenotype suggests a common mechanism underlying this phenotype.

### tp53 is upregulated in mutants for cell-essential genes

Previously, a number of isolated reports showed p53 upregulation in response to deficiencies of some genes, which can be classified as housekeeping. For example, mutations in several genes involved in DNA replication and repair were associated with p53 upregulation [8], [9]. We have recently found that zebrafish mutants for ribosomal proteins (RPs) *rps8*, *rps11*, *rps18*, and *rpl11* have upregulation of the p53 network [10], [11]. Mutations in some other genes involved in ribosomal biogenesis also led to p53 upregulation [12], [13]. Upregulation of p53 was also noted in a mutant for recycling factor p110 [14]. Notably that the phenotype characterized by excessive neuronal apoptosis can be induced in zebrafish by activating p53 through various treatments [15], [16]. Altogether, these data suggest that p53 may mediate non-specific phenotype of mutants for housekeeping genes.

To verify this hypothesis, we examined the p53 network in randomly selected zebrafish mutants from various categories of cell-essential genes. Genes affected in these mutants function in splicing (*u2af1*, small nuclear RNA auxiliary factor), chaperoning (*hspa8*, heat shock protein 8), endocytosis (*clint1*, clathrin interactor 1), ribosome biogenesis (*heatr1*, HEAT repeat containing, BAP-28 like), DNA replication (*top2a*, DNA topoisomerase II-alpha), telomere maintenance (*terfa*, telomeric repeat binding factor a), transcription (*snw1*, SNW domain containing 1), and transport (*atp1a1*, ATPase, Na+/K+ transporting, alpha 1 polypeptide). We found *tp53* upregulation in all these mutants.

**Figure 1:**
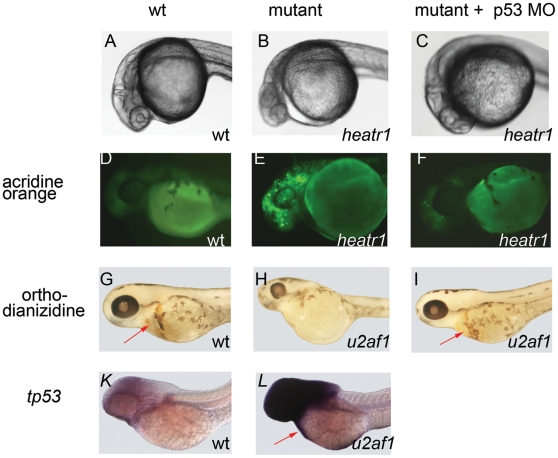
Phenotype of mutants for housekeeping genes are similar and depend on p53 network upregulation. **A–C**) Excessive neural apoptosis was manifested as gray areas in the brain at 24 hpf, which was rescued by p53 inhibition **D–F**) At 48 hpf, there were more apoptotic cells in mutants compared to siblings and p53 inhibition led to a decrease in apoptosis. Acridine orange staining. **G–I**) There were fewer erythroid cells in mutants compared to siblings, which was rescued by p53 inhibition. o-dianizidine staining, 84 hpf, arrow points to erythrocytes. **K–L**) *tp53* upregulation was higher in the brain and blood cells, 48 hpf, in situ hybridization, arrow points to erythrocytes.

The mutants had similar phenotype with prominent neuronal apoptosis as shown by example of *heatr1* mutant. At 24 hours post fertilization, mutants' brains had dark cloudy areas with blurred midbrain-hindbrain boundaries (**Fig. 1A,B**). Acridine orange staining showed that at 48 hpf, when wild-type embryos have very few apoptotic cells, such cells were abundant in mutants, especially in brains (**Fig. 1D,E**). The other tissue affected in the mutants was blood; the mutants had fewer blood cells at 72 hpf (**Fig. 1G,H**). In accord with these data, *tp53* was upregulated the most in neural and blood cells (**Fig. 1K,L**).

Inhibition of p53 by a morpholino prevented neural apoptosis in the mutants (**Fig. 1C,F**). The anemia in the mutants was also rescued by p53 inhibition (**Fig. 1I**). These data suggest that phenotypic changes observed in the mutants were in large part mediated by p53.

**Figure 2:**
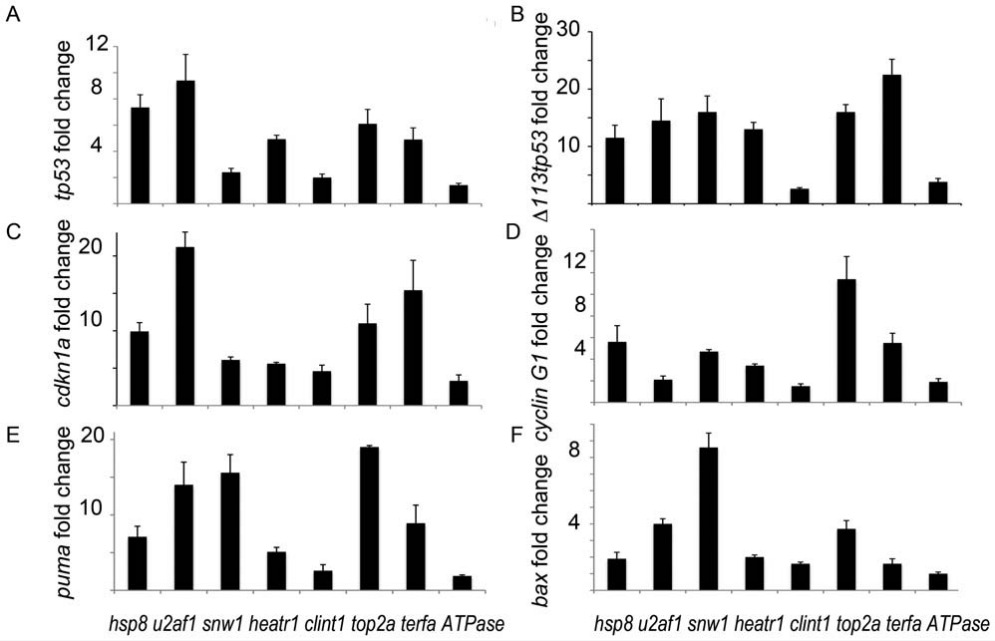
*tp53* and its targets were upregulated in all studied mutants. **A**) The level of *tp53* upregulation varied between mutants. **B**) Δ*113tp53* is a p53 target and p53 inhibitor, a part of p53 autoregulatory loop. **C–D**) Cell cycle suppressor *cdkn1a (p21*) and *cyclin G1* were upregulated. **E–F**) Pro-apoptotic *puma* and *bax* were upregulated in mutants. 48 hpf, qPCR.

The levels of p53 upregulation varied between mutants. At 48 hpf, the strongest upregulation was observed in the *u2af1* mutant, followed by *hsp8* and *top2a* mutants (**Fig. 2A**). Along with the upregulation of full-length *tp53*, we noted an upregulation of a truncated isoform, Δ*113p53* (**Fig. 2B**). This isoform is a part of the p53 autoregulatory loop, it is p53 target and p53 inhibitor. Expression of this isoform is a sensitive indicator of the activated p53 network.

### Mutants have similar changes in several p53-dependent and independent molecular pathways

Among p53-dependent pathways, the most important are those mediating cell cycle arrest and apoptosis. In all mutants included in this study, p53 targets responsible for cell cycle arrest such as *cdkn1a (p21*) and *ccng1 (cyclin G1*) were upregulated (**Fig. 2C,D**) as well as pro-apoptotic p53 targets such as *puma* and *bax* (**Fig. 2E,F**). The activation of p53 targets was not directly proportional to the upregulation levels of *tp53*. For example, cell cycle suppressor *p21* was upregulated the most in the *u2af1* and *terfa* mutants, while pro-apoptotic *puma* was upregulated the most in the *top2a* and *snw1*. Therefore phenotypic changes induced in these mutants by upregulation of these p53 targets would be similar but not identical.

**Figure 3:**
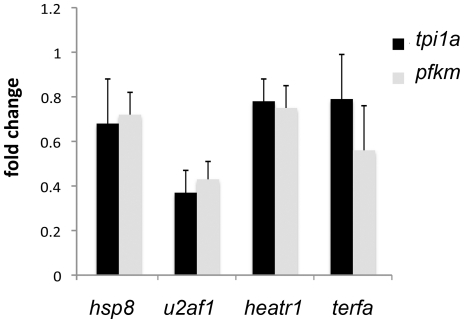
The mutants had shift in energy production to aerobic respiration with supression of glycolysis. Examples in changes in expression of *6-phosphofructokinase, muscle (pfkm)* and *triosephosphate isomerase 1a(tpi1a)* are shown. 48 hpf, qPCR.

Another p53-dependent change found in the mutants examined in this study was alteration in metabolism. It was manifested in a shift in energy production from glycolysis to aerobic respiration; the expression of glycolytic enzymes was suppressed (**Fig. 3**) This observation is in accordance with cell lines data in which the ratio of ATP produced by glycolysis versus aerobic respiration depended on p53 dosage [17]. We also found shift from glycolysis to aerobic respiration in the *rpl11* mutant [11]. Increase in aerobic respiration may lead to an increase in ROS production. In addition, enzymes detoxifying ROS such as catalase, were downregulated in the mutants (data not shown). Therefore, mutants with p53 upregulation are likely to suffer from oxidative stress.

**Figure 4:**
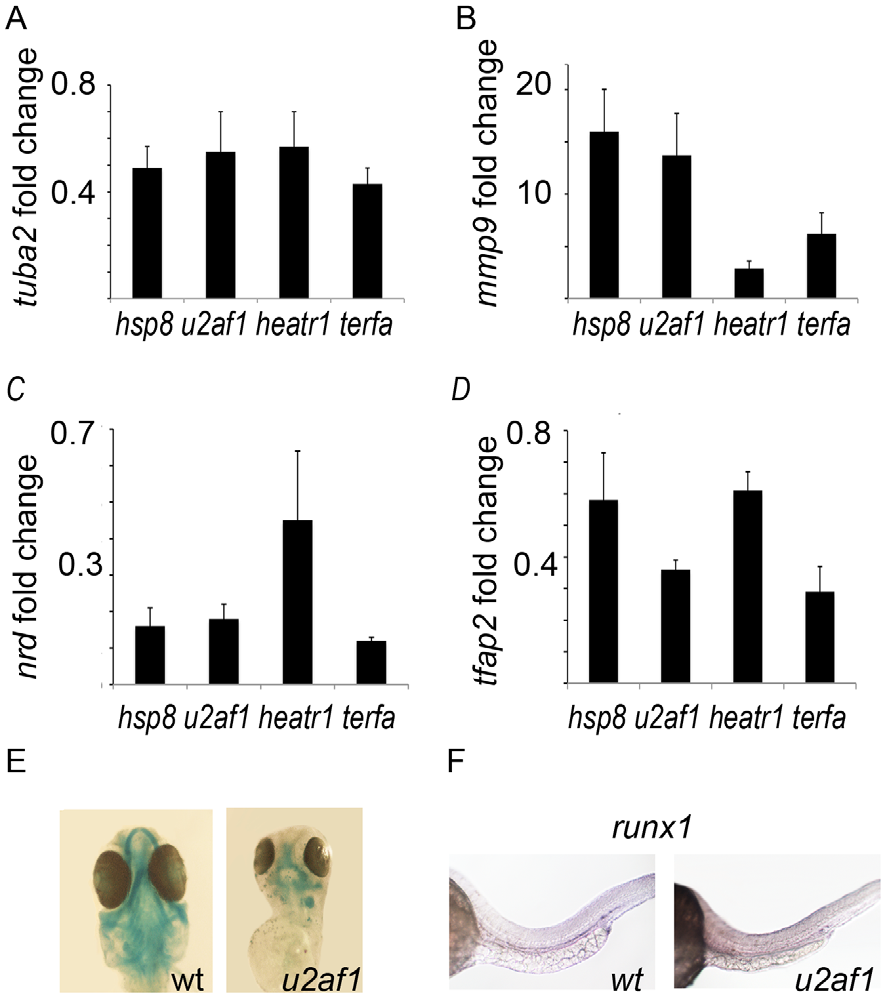
In mutants, biosynthesis was generally suppressed and catabolism was activated. **A**) Expression of structural proteins was downregulated; *tuba2* is shown as an example. **B**) Expression of catabolizing enzymes was upregulated; *mmp9* is shown as an example. **C**) Expression of neural genes was especially strongly suppressed in mutants; *nrd* is an example of a downregulated neural gene. **D**) Expression of a neural crest cell marker *tfap2* was downregulated. **E**) Only rudiments of the head skeleton derived from neural crest cells were present in the mutants. Alcian blue. Day 4. **F**) Upregulation of p53 affects blood development; HSC marker *runx1* was downregulated in the mutants; *u2af1* mutant is shown as an example.

Another important metabolic change noted in the mutants with p53 activation was downregulation of biosynthesis and activation of catabolism. This affected many aspects of cellular homeostasis. For example, a structural protein *tuba2* was downregulated (**Fig. 4A**) while *mmp9* enzyme was upregulated (**Fig. 4B**). The *rpl11* mutant had similar changes [11].

Neural genes such as *nrd* were especially strongly suppressed (**Fig. 4C**). They included genes specific for the neural crest cells, such as *tfap2* (**Fig. 4D**). Accordingly, the mutants formed only rudiments of structures such as the craniofacial skeleton that originates from the neural crest cells (**Fig. 4E**). The primitive wave of blood cells formed normally in mutants, later, however, mutants lost these cells (**Fig. 1G,H**) and the development of the definitive wave of blood cells was suppressed as it was illustrated by the decreased number of cells expressing *runx1*, a marker for hematopoietic stem cells (**Fig. 4F–G**).

**Figure 5:**
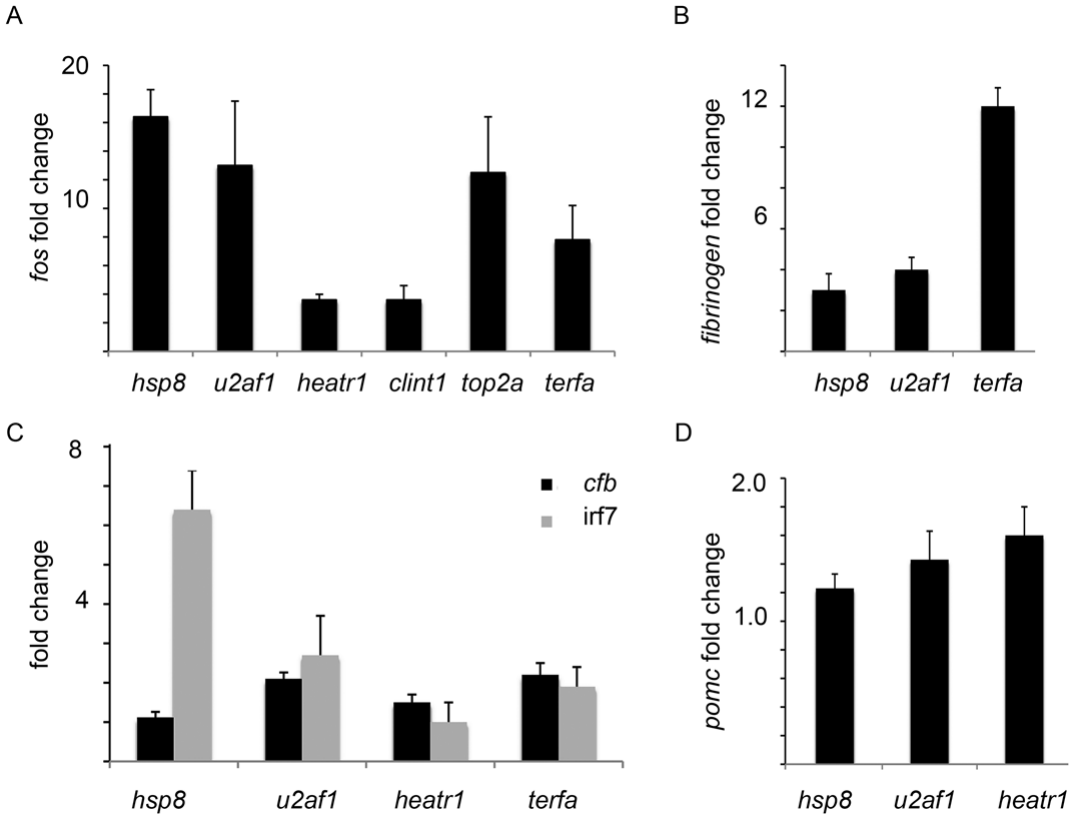
In addition to p53, other stress response pathways were activated in the mutants. **A**) *fos*, a component of AP-1 factor was upregulated. **B**) *fibrinogen* involved in acute phase response was upregulated. **C**) Innate immune mechanisms involving complement and interferon systems were upregulated. **D**) *pomc* that encodes precursor of peptides involved in hypothalamo-pituitary-adrenal hormonal axis response was upregulated.

In addition to the p53 network, other stress response pathways became activated in the mutants. They include immediate early genes such as *fos*, a component of the AP1 factor functioning in the JNK/p38 stress response pathway (**Fig. 5A**), genes involved in the acute phase response such as *fibrinogen* (**Fig. 5B**), and the interferon and complement systems (**Fig. 5C**). Activation of the stress response also took place at the hormonal level. The pituitary responds to stress by increasing the production of pro-opiomelanocortin (Pomc) precursor polypeptide, which gives rise to several peptides including ACTH (**Fig. 5D**).

## Discussion

We analyzed the status of the p53 network in various mutants for cell-essential genes. The genes affected in these mutants function in diverse cellular processes, yet their disruption universally leads to p53 upregulation. It is known that p53 can be activated by various stress signals. p53 response to DNA damage was especially thoroughly studied. Deficiency of several factors involved in DNA replication and repair such as DNA ligase IV was shown to lead to embryonic lethality mediated by p53 pathway [8]. Recently, we and others demonstrated p53 involvement in embryonic death caused by deficiency of ribosomal proteins and other factors involved in ribosome biogenesis. Our study suggests that p53 mediates embryonic lethality caused by deficiency of genes involved in many more basic cellular functions from chromatin maintenance to splicing, endosome/exosome function, chaperoning, and transport.

**Figure 6:**
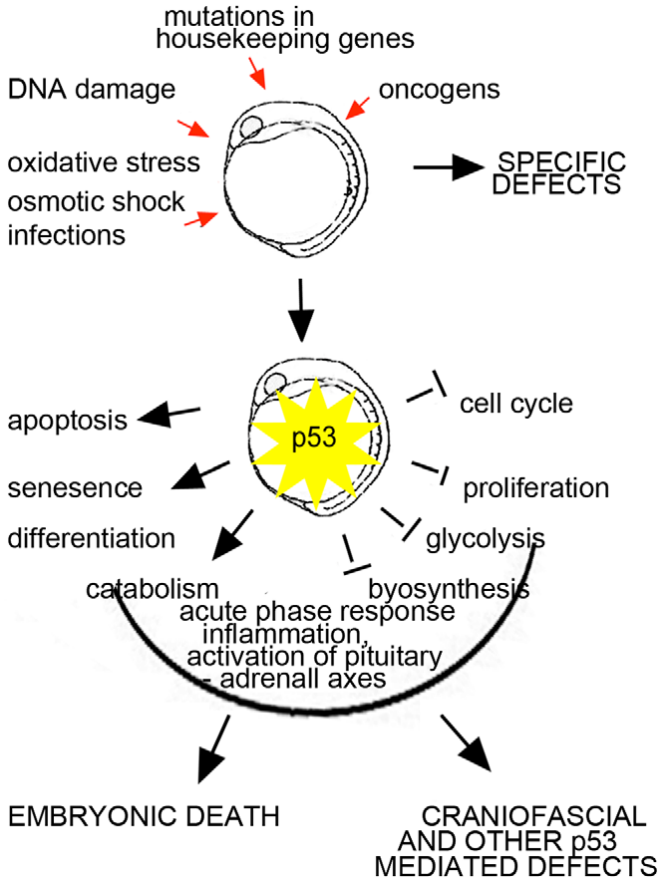
p53 integrates environmental and genetic information into a developmental checkpoint. Each genetic alteration would lead to specific changes in gene expression but changes mediated by p53 may have a major impact on the resulting phenotype. p53 activation can lead to embryonic death or malformations, therefore p53 may in most cases prevent teratogenesis but induce it in other cases. The outcome would depend on the degree of p53 activation, type of stressor and the age and condition of the embryo.

Altogether, these data supports the idea of a p53-based developmental checkpoint. It seems that this checkpoints can be triggered by both genotoxic factors and genetic defects (**Fig. 6**).

p53 is highly expressed in the early embryo [15], [18], which correlates with the high sensitivity of embryonic cells to stressors. Irradiation at doses that have no effects in adult tissues cause p53-dependent apoptosis in embryonic cells [19]. Embryos are especially hypersensitive during gastrulation. In general, maximal p53 activation was found in rapidly proliferating, non-differentiated cells [18]. As the embryo becomes older, p53-mediated response to stressors becomes weaker and more tissue-specific. Blood and hair follicles preserve high p53 responsiveness in adulthood [20].

Anti-teratogenic function of p53 in embryos seems to be a part of a broader function of p53 and members of p53 family p63 and p73 in controlling fidelity of reproduction. It ranges from preserving the integrity of germ cells to regulation of implantation [21], and to early embryonic development.

Apoptotic death in response to p53 activation is not the only possible result of p53 activation in the embryo. In general, the outcome of p53 activation such as apoptosis, senescence, or cell cycle arrest depends on many factors including p53 dosage and the type of cellular defect [22]. The choice between irreversible senescence and reversible quiescence depends on p53 levels and involves mTOR pathway so that inhibition of mTOR promotes quiescence [23], [24]. Haploinsufficiency of the mouse *Rps6* gene leads to the impairment of ribosome biogenesis, high p53 activation and early embryonic death [25]. In contrast, some hypomorphic mutations in ribosomal proteins that cause lower degree of p53 activation are not embryonically lethal [26], [27]. In fact, in the *Rpl24*-deficient mouse embryos, p53 plays pro-survival role, which was suggested to depend of p53 targets inducing cell cycle arrest [27]. mTOR inhibitor *Sestrin 2* was among strongly upregulated genes in *Rpl24*-deficient embryos, which could promote quiescence in embryos helping them to pass embryonic stages at slower pace decreasing the level of cellular stress.

How is the p53-dependent developmental checkpoint activated in embryos? A distinctive feature of early development is rapid cell division, which takes about 15 minutes in zebrafish. Defects in cell-essential genes slowing the embryo's progression through development may lead to increased mitogenic signaling. Indeed, such increase was noted in the *rpl11* mutant [11]. Enhanced delivery of growth factor may lead to stalling of DNA replication forks and double strand breaks (DSBs) [28]. Mitogens would activate the Arf-p53 axis, while DSBs would signal to p53 through ATM (ataxia telangiectasia mutated). In support, cells deficient in telomeric repeat binding factor TRF2 activate p53 through ATM signaling [29]. Moreover, in vitro depletion of splicing factor ASF/SF2 also resulted in DSBs [30]. However, genetic inactivation of *p19Arf* in RPS6-deficient mouse embryos does not rescue the embryos and these embryos do not activate ATM suggesting other pathways mediate p53 upregulation in this model [25]. Therefore, the connection between cellular oncogenes and p53 activation in mutants for housekeeping genes needs further investigation.

Another idea is that the nucleolus serves as a universal stress sensor mediating p53 activation [31]. Some data support this idea, while others do not [32]. Instead, activation of p53 in response to defects in ribosome biogenesis was suggested to depend on RPs not incorporated into ribosomes. Some RPs bind Mdm2 and activate p53 in this way. All of these mechanisms, however possible, do not explain the transcriptional upregulation of p53. Among possible p53-transactivating factors are AP1 factor genes such as *fos, myc* family, and NFkB. p53 promoter carries AP1-binding element, Myc E-box DNA binding motif, and NFkB binding site [33]. Involvement of other factors stabilizing *tp53* mRNA also cannot be excluded [34].

It is also possible that p53 activation in embryos with genetic defects is a cumulative effect of several checkpoints merging at p53. In any case, although p53 is acting at the cellular level, it may ultimately provide an integrating checkpoint at the organism level by preventing embryos with genetic defects, damaged, or stressed from developing beyond the initial stages. The role of p53 at the population level may indeed be similar to its “guardian of the genome” role at the cellular level, where it removes defective cells from the proliferating pool.

Our data suggest that mutations in hundreds of housekeeping genes may result in the upregulation of the p53 network. This means that the classic genotype – phenotype concept cannot be applied to mutations in many housekeeping genes since the resulting phenotype would depend on p53. In fact, many papers have interpreted the phenotypic changes in mutants for housekeeping genes as a proof that these genes are directly involved in the development of a particular tissue. Especially alterations in the development of brain, neural crest derivatives, and blood, which are the most sensitive to p53 upregulation, can be easily misinterpreted. Therefore, genotype-phenotype mapping should take into account that a large proportion of genes would canalize on a p53-dependent phenotype. The upregulation of p53 in response to a deficiency of housekeeping genes may also account, at least partially, for the phenomenon of frequent p53 activation by various knockdown techniques [Robu, 2007 #2668].

While p53 seems to be a universal anti-teratogenic factor, there is another side of p53. If, for any reason, embryos experience p53 upregulation but survive, they may carry the malformations caused by p53 upregulation. Neural and blood cells are the most sensitive targets. An example of p53-mediated pathology in humans is Treacher Collins syndrome, which is caused by p53-mediated apoptosis in neural crest cells [Jones, 2008 #3375].

p53 activation during early development induced by various stressors may be the reason why human congenital anomalies caused by diverse factors are similar. The p53 network may have a much more important role in the origin of human congenital malformations than it is currently appreciated. In the future, evaluation of the p53 family status may become an informative tool in prenatal diagnostics.

## Materials and Methods

### Ethics Statement

Zebrafish embryos were handled according to relevant national and international guidelines.

UCLA office for protection of research subjects approved the use of zebrafish (ARC # 2001-074-22).

### Zebrafish

The following mutant lines have been used: *u2af1^hi199Tg^*; *hspa8^hi138Tg^*; *clint1^hi 1520Tg^*; *terfa^hi3678Tg^*; *snw1^hi296bTg^*; *top2a* ^hi3635Tg^; *heatr1^hi932Tg^*; *atp1a1^m883/m883^*. Fish of the wild type TU and mutant strains were reared at 28.5°C at a 14-hr light/10-hr dark cycle. Embryos were obtained by natural spawning.

### qRT-PCR

RNA was prepared using Trizol (Invitrogen, Carlsbad, CA) from pool of 30–40 embryos. cDNA was synthesized by reverse transcription of 2 µg of RNA with the random hexamer primers. Quantative PCR (qPCR) was performed using iQ SYBR Green Super Mix and a MyiQ Single-Color PCR thermal cycler (Biorad, Hercules, CA). Each experiment was performed in triplicate. Levels of mRNA expression in mutants relative to sibling controls were normalized to beta-actin and calculated according to the Cτ method. Primer sequences are available upon request.

### In situ hybridization

Whole mount in situ hybridization was carried out as described [35] with *tp53* and *runx1* riboprobes.

### Morpholinos

To inhibit p53, a previously described p53 morpholino 5′-gcgccattgctttgcaagaattg, inhibitor of translation, was used [15] (Gene Tools, Corvallis, OR). The 3ng of morpholino was injected at the one cell stage.
